# Supplementary material for: Giant narrowband twin-beam generation along the pump-energy propagation direction
Source: Nat Commun. 2015 Jul 17;6:7707. doi: 10.1038/ncomms8707 (PMC4518286; doi:10.1038/ncomms8707)
Supplement: Supplementary Information — Supplementary Figures 1-3, Supplementary Methods and Supplementary References [file ncomms8707-s1.pdf]

I. SUPPLEMENTARY FIGURES

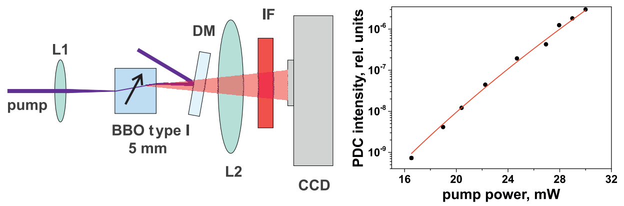

Supplementary Figure 1. Left panel: experimental setup for obtaining 2D angular spectra of PDC. The pump radiation is focused into the crystal by means of lens L1 with the focal length 500 mm. The optic axis direction is shown by an arrow. After the pump is cut off with a dichroic mirror DM, PDC radiation is collected by lens L2 with the focal distance 26 mm and NA 0.5 and filtered by an interference filter IF (bandwidth 10 nm, central wavelength 710 nm). A CCD camera is then placed into the focal plane of the lens L2. Right panel: the dependence of the PDC intensity on the pump power, from which the gain value can be obtained. Solid line is a fit with Eq. (1).

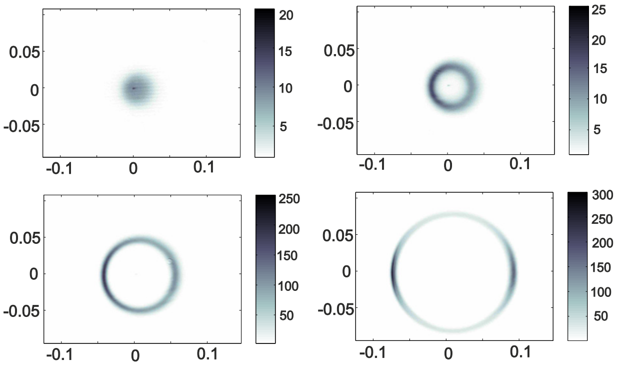

Supplementary Figure 2. Two-dimensional spectra of PDC obtained at crystal orientations 32.97°, 33.07°, 33.32°, and 33.95°. The axis labels correspond to external angles of emission in radians. Note that the color code is different in all plots.

II. SUPPLEMENTARY METHODS

The setup for the experiment with the transverse walk-off is shown in Supplementary Figure 1. As a pump, we used third-harmonic radiation of a YAG:Nd laser, with the wavelength 355 nm, pulse duration 18 ps, and the repetition rate 1 kHz. The pump was focused into the crystal by means of lens L1 with the focal length 500 mm, which created a waist with the full width at half maximum (FWHM) 60 μm. The BBO crystal of length 5 mm was tilted to reach different kinds of type-I phasematching. For instance, collinear emission at the degenerate

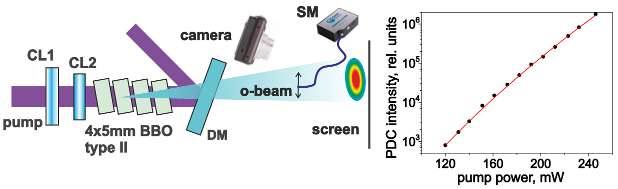

Supplementary Figure 3. Left panel: Experimental setup for the study of giant twin-beam generation under group-velocity matching. The pump is focused into the crystals by means of a telescope made of two cylindrical lenses CL1, CL2. In the plane of the optic axes of the crystals, the beam is unfocused. The pump is cut off with a dichroic mirror DM, and the ordinary PDC radiation is either observed on a screen or analyzed by means of a fibre spectrometer SM. Right panel: the dependence of the PDC intensity on the pump power in case of four crystals, from which the gain value can be obtained. Solid line is a fit with Eq. (1).

wavelength 710 nm requires the orientation 32.97° to the optic axis, while the orientation at which the cone of emission at 710 nm contains the pump Poynting vector direction is 34.9°.

For capturing the angular spectra, an aspheric lens L2 with the focal distance $f = 26$ mm was placed at a distance 30 mm from the crystal, after an interference filter IF with the bandwidth 10 nm and the central wavelength 710 nm. The large numerical aperture (NA 0.5) allowed us to collect all range of interest for external angles of emission. In the focal plane of the lens, a CCD camera was placed, so that it recorded the 2D angular intensity distribution. The coordinate on the camera x corresponded to the external angle of emission θ as $x = f \tan\theta$. Four intensity angular distributions, for the crystal orientations 32.97°, 33.07°, 33.32°, and 33.95°, are shown in Supplementary Figure 2. We see that as the angle of emission approaches the walk-off angle, the emission is strongly enhanced for two directions, the one along the pump Poynting vector and the other one, along the phase-matched wavevector. One can notice the asymmetry between the left and right sides of the rings: the left side has higher peak intensity but smaller width. This is caused by the anisotropy [1, 2].

The parametric gain was found by measuring the output PDC intensity I as a function of the pump power P and fitting the obtained dependence (Supplementary Figure 1, right panel) by the equation

$$I = A \sinh^2(B\sqrt{P}), \tag{1}$$

with A, B being the fitting parameters. The parametric gain at a given pump power P is calculated as $G = B\sqrt{P}$ [3]. For the case of emission along the pump Poynting vector, the parametric gain was found to be $G = 15$.

For the experiment with the longitudinal walk-off, we used shorter pulses produced by frequency-doubled radiation of a Mai Tai/Spitfire system with the wavelength 400 nm, pulse duration 1.2 ps, repetition rate 5 kHz and

mean power up to 700 mW. The experimental setup is shown in Supplementary Figure 3. The pump was focused into type-II BBO crystals by means of a telescope consisting of two cylindrical lenses, in order to keep the beam wide enough in the plane of the optic axis and to minimize the effect of spatial walk-off. The FWHM of the beam in the plane of the optic axes was 3.4 mm. To be able to change the length of the nonlinear crystal, we used either four BBO crystals of length 5 mm stacked together or a single such crystal. The pump was focused into the crystals differently: with a lens of focal length 500 mm in the case of a single crystal and with a 30:2.5 telescope in the case of four crystals. This difference is due to the fact that more tight focusing is required in a shorter crystal, which, in its turn, leads to a Rayleigh length too small for the 4-crystal stack. At the same time, measurements were performed at the same parametric gain ($G = 8.6$).

The four crystals were stacked together with the minimal spaces between them (3 mm). After the crystals, the pump radiation was cut off by a dichroic mirror and the PDC radiation was either observed on a screen placed at $d = 76$ cm from the samples or analyzed by means of an Ocean Optics HR4000 fibre spectrometer. The screen had a ruler pasted to it, so that the external angles of emission θ could be determined from the coordinate X on the screen as $\theta = \arctan(X/d)$. The spectra on the screen were captured by a photographic camera. Alternatively, by placing the spectrometer input (a multimode fibre with the diameter 400 μm) at a distance 87 mm from the crystals and scanning it in the direction orthogonal to the pump propagation (shown by an arrow), we could measure wavelength spectra at various angles. Note that in type-II PDC, the ordinary (o) cone of radiation is tilted with respect to the extraordinary (e) one. Due to this, we were able to study only the o-beam without overlapping with the e-beam.

To describe the obtained intensity distributions theoretically, we applied our model described in detail in Ref. [4] and used in Ref. [5]. Because the anisotropy manifests itself significantly only in the plane of the optic axis, the Hamiltonian can be calculated using only one dimension in the Cartesian frame of reference [4]:

$$H = i\hbar\Gamma \int d\vartheta_s d\vartheta_i F(\vartheta_s, \vartheta_i) a^\dagger_{\vartheta_s} a^\dagger_{\vartheta_i} + h.c., \qquad (2)$$

where Γ is the coupling parameter, depending on the quadratic susceptibility, pump amplitude, and the crystal length, $\vartheta_{i,s}$ are the angles of emission of idler and signal photons inside the crystal, related to the external angles and the Cartesian coordinates in the far field as shown in subsection A, and $a^\dagger_{\vartheta_{i,s}}$ are photon creation operators in the corresponding plane-wave modes. The core of the Hamiltonian, usually called the two-photon amplitude (TPA), in the presence of anisotropy can be written as [2]

$$F(\vartheta_s, \vartheta_i) = \exp[-\frac{\sigma^2(\Delta_\perp \cos\rho + \Delta_\parallel \sin\rho)^2}{2}] \exp[-i\frac{L}{2}\xi]\mathrm{sinc}[\frac{L}{2}\xi], \qquad (3)$$

where $2\sqrt{ln2}\sigma$ is the FWHM of the pump spatial intensity distribution, ρ is the walk-off angle, $\Delta_\parallel = k_p - k_s\cos\vartheta_s - k_i\cos\vartheta_i$ is the longitudinal mismatch, $\Delta_\perp = k_s\sin\vartheta_s + k_i\sin\vartheta_i$ is the transverse mismatch, L is the crystal length, and $\xi = \Delta_\parallel - \Delta_\perp\tan\rho$.

The Schmidt decomposition of the TPA has the form

$$F(\vartheta_s, \vartheta_i) = \sum_n \sqrt{\lambda_n} u_n(\vartheta_s) v_n(\vartheta_i), \qquad (4)$$

where λ_n are the Schmidt eigenvalues and $u_n(\vartheta_s)$, $v_n(\vartheta_i)$ the Schmidt eigenfunctions [6]. By passing to collective photon creation operators $A_n^\dagger, B_n^\dagger$ corresponding to the Schmidt modes in the angular spectrum,

$$A_n^\dagger = \int d\vartheta_s u_n(\vartheta_s) a^\dagger_{\vartheta_s},$$
$$B_n^\dagger = \int d\vartheta_i v_n(\vartheta_i) a^\dagger_{\vartheta_i}, \qquad (5)$$

we diagonalize the Hamiltonian (the Bloch-Messiah reduction),

$$H = i\hbar\Gamma \sum_n \sqrt{\lambda_n}(A_n^\dagger B_n^\dagger - A_n B_n). \qquad (6)$$

Further, we write time-depending differential equations for the new operators in the Heisenberg representation,

$$\frac{dA_n}{dt} = 2\Gamma\sqrt{\lambda_n}B_n^\dagger, \frac{dB_n^\dagger}{dt} = 2\Gamma\sqrt{\lambda_n}A_n, \qquad (7)$$

and find their solutions given by the Bogolyubov transformations,

$$A_n^{out} = A_n^{in}\cosh[\tilde{G}\sqrt{\lambda_n}] + [B_n^{in}]^\dagger \sinh[\tilde{G}\sqrt{\lambda_n}],$$
$$B_n^{out} = B_n^{in}\cosh[\tilde{G}\sqrt{\lambda_n}] + [A_n^{in}]^\dagger \sinh[\tilde{G}\sqrt{\lambda_n}], \qquad (8)$$

where $\tilde{G} \equiv \int 2\Gamma dt$ and A_{mn}^{in}, B_{mn}^{in} are the initial (vacuum) photon annihilation operators. Finally, the equations for the plane-wave photon creation operators can be obtained and solved analytically in the Heisenberg picture using the expressions for the Schmidt-mode photon creation operators. Then the mean photon number in the signal beam can be calculated by averaging over the vacuum state,

$$\langle N_s \rangle = \sum_n |u_n(\vartheta_s)|^2 (\sinh[\sqrt{\lambda_n}\tilde{G}])^2, \qquad (9)$$

and similarly for the idler beam. Thus, the total intensity distribution results from incoherent contributions of Schmidt modes with new weights that differ dramatically from the initial Schmidt eigenvalues λ_n and strongly

depend on the parametric gain. The renormalized new weights of different Schmidt modes are

$$\Lambda_n = \frac{(\sinh[\tilde{G}\sqrt{\lambda_n}])^2}{\sum_n (\sinh[\tilde{G}\sqrt{\lambda_n}])^2}. \quad (10)$$

At high gain, the mode corresponding to the Poynting vector direction becomes much more pronounced than the others because its eigenvalue is dramatically enhanced.

III. SUPPLEMENTARY REFERENCES

[1] Cavanna, A. *et al.* Compensation of anisotropy effects in the generation of two-photon light. *Optics Express* **22**, 9984 – 9992 (2014).

[2] Perez, A. *et al.* Compensation of anisotropy effects in a nonlinear crystal for squeezed vacuum generation. *Laser Physics Letters* **10**, 125201 – 125206 (2013).

[3] Agafonov, I. N., Chekhova, M. V. & Leuchs, G. Two-color bright squeezed vacuum. *Phys. Rev. A* **82**, 011801 (2010).

[4] Sharapova, P. R., Pérez, A. M., Tikhonova, O. V., & Chekhova, M. V. Schmidt modes in the angular spectrum of bright squeezed vacuum. *Phys. Rev. A* **91**, 043816 (2015).

[5] Pérez, A. M. *et al.* Bright squeezed vacuum source with 1.1 spatial mode. *Optics Letters* **39**, 2403 – 2406 (2014).

[6] Miatto, F. M., Di Lorenzo Pires, H., Barnett, S. M. & van Exter, M. P. Spatial Schmidt modes generated in parametric down-conversion. *Eur. Phys. J. D* **66**, 263 (2012).
